# Molecular-Scale Interactions in the Choline Chloride–Ethylene Glycol Deep Eutectic Solvent System: The Importance of Chromophore Charge in Mediating Rotational Dynamics

**DOI:** 10.1021/acs.jpcb.4c04118

**Published:** 2024-09-24

**Authors:** Allison Stettler, Piyuni Ishtaweera, Gary A. Baker, Gary J. Blanchard

**Affiliations:** † Department of Chemistry, Michigan State University, East Lansing, Michigan 48824-1322, United States; ‡ Department of Chemistry, University of Missouri-Columbia, Columbia, Missouri 65211, United States

## Abstract

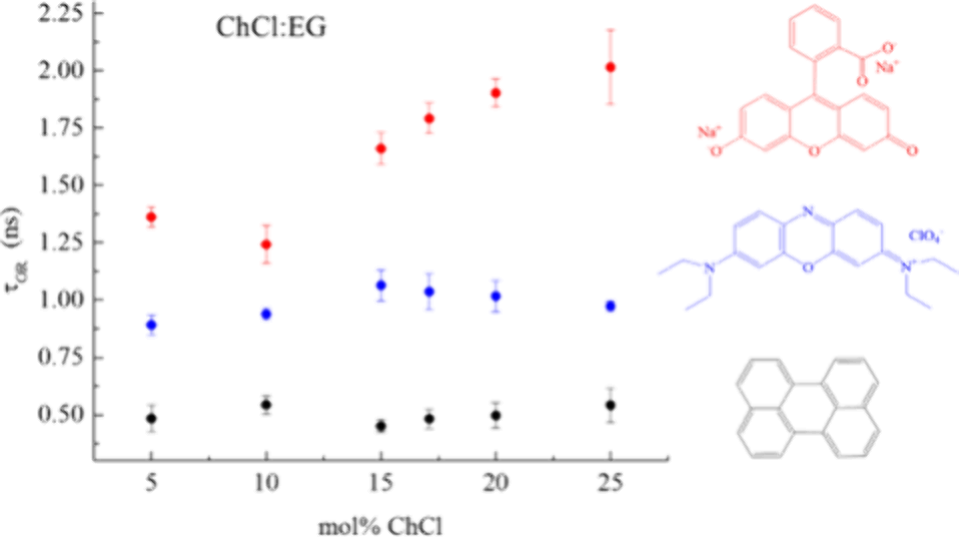

We report on the rotational diffusion dynamics of three chromophores (disodium fluorescein, oxazine 725, and perylene) in a series of choline chloride–ethylene glycol (ChCl:EG) deep eutectic solvent (DES) systems. We observe behavior independent of DES bulk viscosity for the cationic and neutral probes and behavior that is consistent with stick-limit interactions for the modified Debye–Stokes–Einstein model for the anionic probe. This finding indicates that the anionic species is integral to the interactions between DES constituent species that are responsible for local organization, consistent with previous MD simulations that showed higher interaction energies associated with both the hydrogen bond donor (EG) and hydrogen bond acceptor (Ch^+^) interactions with Cl^–^ in ChCl:EG mixtures. The reorientation data reported here also indicate a region around 15 mol % ChCl where the stoichiometric relationship between the species gives rise to changes in the details of intermolecular interactions.

## Introduction

Developing green approaches to pre-existing and future industrial needs is an issue that has generated a great deal of interest in both academic and industrial spheres over the past several decades. Many current processes rely on harsh organic solvents that are volatile, toxic, and difficult to dispose of, resulting in significant environmental pollution.^1−3^ Several solutions to this problem have been proposed, with ionic liquids (ILs) and deep eutectic solvents (DESs) emerging as potential solutions.

ILs and DESs have garnered much attention due to their low volatility, low flammability, and high tunability.^4,5^ While ILs and DESs have been proposed as solvents for similar applications, an ongoing issue with ILs is the toxicity of synthetic IL components, which make applications in pharmaceutical and food industries difficult.^6,7^ This issue, along with the lack of biodegradability, have made DESs a much more attractive solution to make preexisting processes greener.^6^

DESs represent a fascinating class of increasingly useful solvents that have potential applications in biomass processing, pharmaceutical research, CO_2_ capture, redox flow batteries, nanotechnology, organic synthesis, environmental remediation, and metal processing.^5,8−15^ These solvent systems are composed of two or more compounds that produce, upon mixing, a free-flowing fluid that has a melting point significantly lower than any of its neat constituents. Unlike ILs, source materials for DESs are generally relatively inexpensive, sustainable, biodegradable, and nontoxic.^4,5^ In addition, DESs display higher tunability relative to ILs as properties of DESs can be changed simply by altering molar ratios of components, substituting one constituent of the mixture, or adding cosolvents.^4,5,10,16^ Further, these mixtures are easy to prepare, often at 100% atom efficiency, and require few, if any, purification steps. The popular Type III DESs consist of two components: a hydrogen bond acceptor (HBA) and a hydrogen bond donor (HBD). HBAs in DESs are most commonly quaternary ammonium salts with HBDs consisting of amines, amides, alcohols, or carboxylic acids.^4,5^ DES mixtures are also reported to form from sugars, polyols, carboxylic acids, and amino acids.^16−18^ The first reported DES consisted of a mixture of choline chloride (ChCl) and urea in a 1:2 (ChCl:urea) molar ratio.^19^ Hydrogen bonding between HBA and HBD substituents is thought to be the dominant process contributing to the melting point depression in DESs due to the formation of extensive hydrogen bonding networks, with some contribution from electrostatic and van der Waals forces.^20^ As these solvents are relatively recent discoveries, there is a lack of cohesive and comprehensive knowledge about the specific intermolecular interactions that characterize DES mixtures. It is therefore imperative that solute–solvent interactions in these mixtures be investigated and understood. A major issue impeding the elucidation of the structural details of DES mixtures is their characteristically high viscosity. This is thought to be due to an extensive hydrogen bonding network between HBAs and HBDs. Previous research has focused on ethaline—the canonical DES comprising a 1:2 molar mixture of choline chloride (ChCl) to ethylene glycol (EG)—as a foundational model to set a benchmark for forthcoming DES investigations.^5,21−24^ The notably low viscosity of ethaline relative to other DES systems distinguishes it as an optimal candidate for study, offering researchers an accessible starting point for probing basic DES characteristics and behaviors. We note that, while ethaline is generally regarded as a typical Type III DES,^25^ the ChCl + EG phase diagram apparently follows the predictions for an ideal binary nonelectrolyte mixture. In fact, Agieienko and Buchner have suggested that a 1:2 molar ratio of ChCl: EG is not the true eutectic composition, which they propose actually lies at 1:4.85 (i.e., *x*_ChCl_ = 0.171 instead of 0.333).^26,27^ This is further supported by the recent work of the Sangoro group, which determined that the fastest orientational and solvation dynamics of ChCl:EG DESs lies within 15–20 mol % ChCl.^28^ It is also important to note that our own visual observations of the 1:2 ChCl:EG DES mixture indicate significant crystallization at 294 K, which is far above the reported eutectic temperature.^28^ To shed some light on this conundrum, it is important to study dynamics within mixtures comprising a wide range of compositions of ChCl and EG.

**Figure 1:**
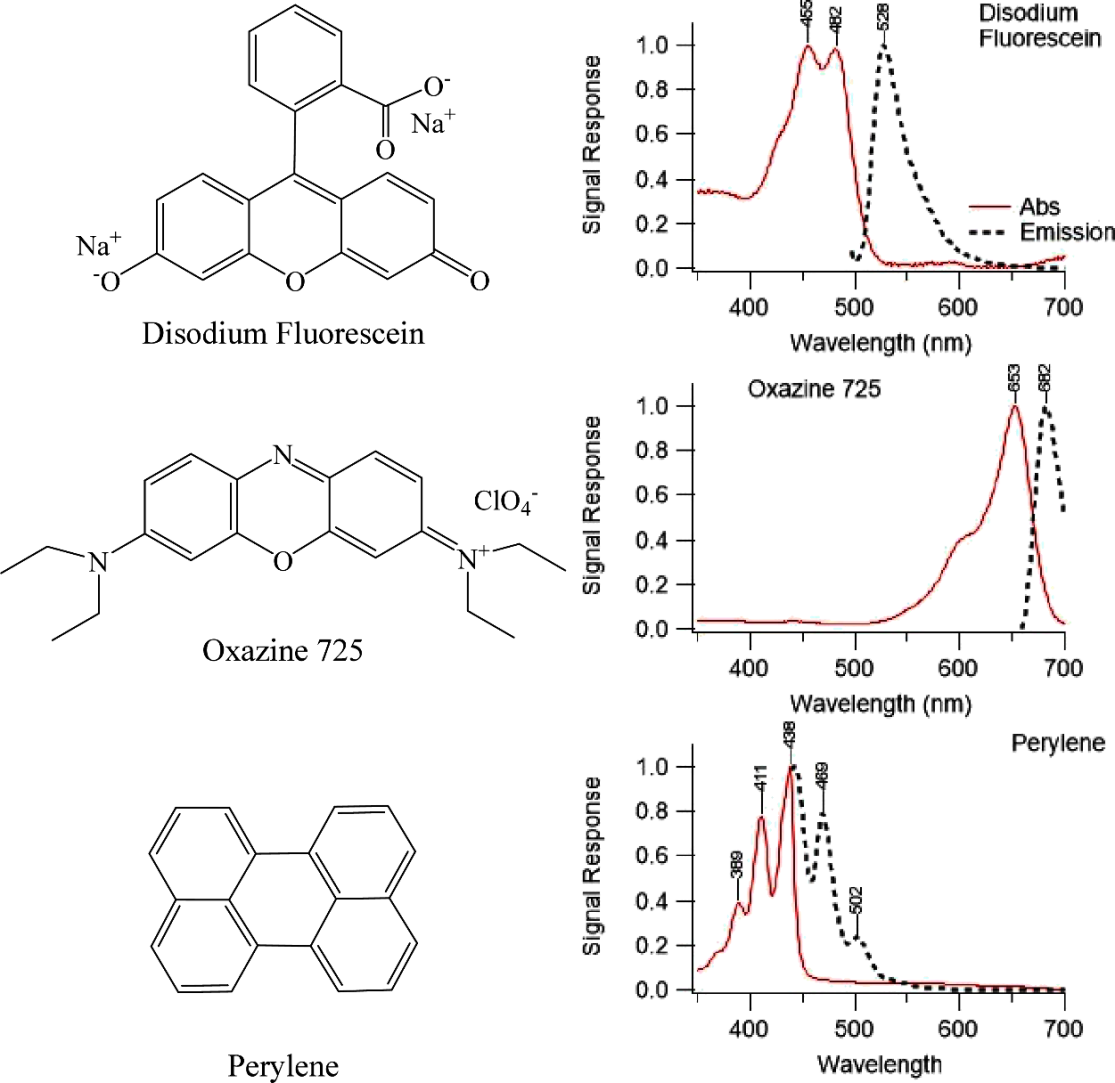
Chemical structures of fluorescent probes and their corresponding absorbance and emission spectra. Solid lines (red) are absorbance spectra; dashed lines (black) are fluorescence emission spectra.

Experimental and theoretical methods have indicated dynamic heterogeneity with no evidence of static heterogeneity in ethaline over a wide temperature range.^29^ Interestingly, previous studies making use of experimental and theoretical methods have indicated that most DESs in general are heterogeneous.^29−34^ Understanding the details of this heterogeneous environment is paramount in determining solute–solvent interactions as this knowledge will define future experimental designs and eventual industrial adoption and scale-up involving DESs. While there have been studies probing the rotational diffusion dynamics of fluorophores in differently charged environments in ethaline over a range of temperatures, to our knowledge, no studies have been conducted probing the rotational diffusion dynamics of differently charged environments across varying molar ratios of the constituent compounds in a DES.^23^ Our aim with this work is to determine whether different solute molecules experience different local environments in DESs and whether those environments depend on DES composition. Specifically, we have made use of both charged (disodium fluorescein (DSF, dianion); oxazine 725 (Ox725, monocation)) and neutral (perylene) fluorescent probes (Figure 1) using time-correlated single photon counting (TCSPC) time-domain fluorescence spectroscopy to probe rotational diffusion dynamics in varying ChCl:EG molar ratio mixture as a measure of heterogeneity. The data reveal several interesting effects that point collectively to the importance of chromophore charge in determining its interactions with these dynamic heterogeneous systems.

## Experimental Methods

### Materials Used

A series of choline chloride (ChCl):ethylene glycol (EG) DESs of compositions 5 mol % ChCl (1:19 ChCl:EG), 10 mol % ChCl (1:9 ChCl:EG), 15 mol % ChCl (1:5.67 ChCl:EG), 17.1 mol % ChCl (1:4.85 ChCl:EG), 20 mol % ChCl (1:4 ChCl:EG), 25 mol % ChCl (1:3 ChCl:EG), and 33 mol % ChCl (1:2 ChCl:EG) were prepared using ChCl (Sigma-Aldrich, BioUltra, ≥99.0%) and EG (Sigma-Aldrich, ReagentPlus, ≥99%) that were used as received. Binary ChCl:EG samples containing 5–33 mol % ChCl were prepared according to Table S1 in the Supporting Information, following methods reported earlier.^35,36^ Appropriate masses of ChCl and EG were weighed to an accuracy of ±0.0001 g on an analytical balance (Mettler Toledo LA204E) within a dry 250 mL round-bottom flask followed by rotary evaporation at 80 °C for 30 min with 100 rpm rotation. The clear, colorless, uniform fluids were transferred to precleaned 40 mL EPA vials (capped with PTFE-faced silicone rubber septa) equipped with PTFE-coated stir bars and further dried overnight at 70 °C under vacuum while stirring. The chromophores disodium fluorescein (DSF, Sigma-Aldrich), oxazine 725 (Ox725, Exciton), and perylene (Sigma-Aldrich) were used as received. Stock solutions of each chromophore in ethanol were made and aliquots of the stock solutions introduced to a volumetric flask, reduced to dryness, and then taken up in the DES of choice. Final chromophore concentrations were 10^–6^ M for all systems examined.

### Time-Correlated Single Photon Counting

The time-correlated single photon counting (TCSPC) instrument used in this work has been described in detail before^37^ and we provide a brief recap here. The source laser is a passively mode-locked diode-pumped Nd:YVO_4_ laser (Spectra Physics Vanguard) that produces 13 ps pulses at a repetition rate of 80 MHz. The average power output at the second harmonic (532 nm) is 2.5 W and, at the third harmonic (355 nm), it is 2.5 W. The outputs of this laser are used to excite two synchronously pumped cavity dumped dye lasers (Coherent 701-3 with 7220 cavity dumpers and 7200 cavity dumper drivers). The dye laser pumped by the third harmonic output of the pump laser was operated with Stilbene 420 laser dye (Exciton) at a wavelength of 435 nm (ca. 10 mW average power at 4 MHz repetition rate, 5 ps pulses) and the dye laser excited by the second harmonic output of the pump laser was operated with Rhodamine 6G laser dye at 575 or 630 nm (ca. 20 mW average power at 4 MHz repetition rate, 5 ps pulses). For all measurements, the vertically polarized excitation pulses were incident on the sample at intensities of less than 1 mW average power. Fluorescence from the sample is collected at right angles to the excitation axis using a 40× reflecting microscope objective (Ealing), split using a polarization-selective cube beam splitter, and sent to two subtractive double monochromators (Spectral Products CM 112) equipped with microchannel plate photomultiplier (MCP-PMT) detectors (Hamamatsu R3809U-50). Each detector output is sent to the TCSPC detection electronics (Becker & Hickl SPC-132). The detector reference channel (Becker & Hickl PHD-400-N photodiode) is excited by a portion of the excitation beam. The TCSPC electronics are controlled by software written in-house using LabVIEW software. The instrument response for this system is ca. 40 ps.

### Steady State Spectroscopies

Absorbance measurements were performed using a Cary 4000 UV–visible spectrometer, with 1 nm spectral resolution for all measurements. Fluorescence measurements were performed with a Spex Fluorolog 3 emission spectrometer, also with 1 nm spectral resolution for all measurements. Commercial software from the instrument manufacturer was used for data acquisition.

## Results and Discussion

The primary purpose of this work is to gain a deeper understanding of local organization and dynamics in DESs. We have chosen the ChCl-EG system because of its wide use and the relatively well-defined range of intermolecular interactions that are thought to proceed in this system.^38^ While perhaps the most widely studied DES system is ethaline (i.e., 1:2 ChCl:EG), the focus of our work is on systems of variable composition to explore solute rotational dynamics across this binary system including the EG-rich region, which shows more favorable viscosities for practical applications. We were also interested in establishing whether recently reported composition-dependent dynamics in these systems^28^ could be resolved using fluorescent probes of different charge and polarity. Our findings, described below, reveal a significant chromophore charge dependence and a DES composition dependence on the observed rotational diffusion dynamics of the chromophores shown in Figure 1.

The extraction of rotational diffusion information from polarized emission transients is well established, with the induced orientational anisotropy decay function, *R*(*t*), being constituted from the raw polarized time-resolved emission data according to eq 1.
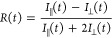
1

The functional form of the *R*(*t*) decay contains the physical and chemical information on interest. *R*(*t*) can contain up to five exponential decays, but in practice, either one or two exponential decays are observed depending on the ellipsoidal shape of the rotor and the relative orientations of the chromophore’s absorption and emission transition dipole moments. For all the data reported here, *R*(*t*) is observed to decay as a single exponential, and the time constant of the decay is related to system properties through the modified Debye–Stokes–Einstein (DSE) equation (eq 2),^39^
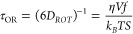
2where *τ*_*OR*_ is the decay time constant of *R*(*t*), *D*_*ROT*_ is the rotational diffusion constant, *η* is the viscosity of the medium surrounding the rotating moiety, *V* is the rotor hydrodynamic volume,^40^*f* is the frictional boundary condition term, *k*_*B*_*T* is the thermal energy of the system, and *S* is a term to describe the ellipsoidal shape of the rotor.^41^ In this model, the frictional interaction term describes the interactions between the chromophore and its immediate environment, where *f* = 1 is the so-called stick limit, representing comparatively strong frictional interactions. The stick limit is typically appropriate for polar, charged chromophores within polar solvent systems. For weaker frictional interactions, more characteristics for the reorientation of nonpolar chromophores in nonpolar environments, the *f* term is described in the slip limit,^42^ implying lower energy intermolecular interactions. The details of slip-limit behavior have been described by Hu and Zwanzig^42^ and the value of *f* depends on the effective rotor shape, S, as calculated by Perrin.^41^ We will consider the experimental data for the three chromophores studied here (fluorescein, oxazine 725, and perylene) in the context of the modified DSE model.

**Figure 2:**
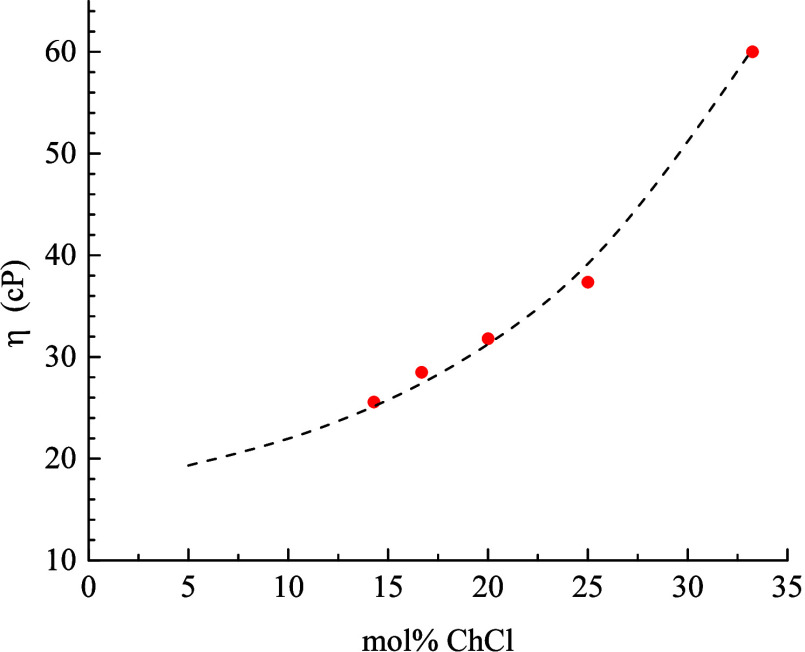
Composition dependence of ChCl:EG DES viscosity. Solid red points are experimental data from ref (21), and the dashed line is the best-fit line of the data to eq 3.


**Figure 3:**
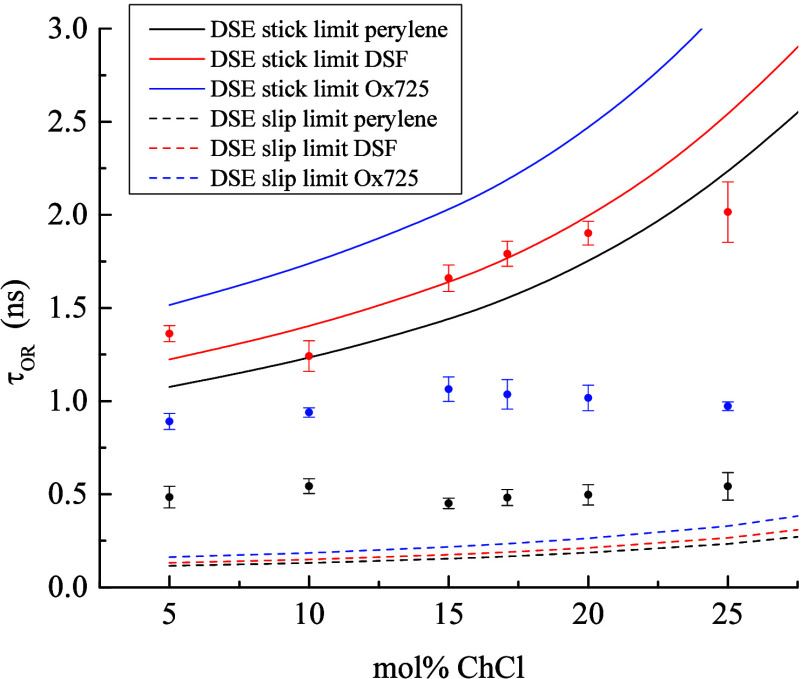
Experimental τ_OR_ data (points) for Ox725 (blue), DSF (red), and perylene (black) as a function of ChCl:EG DES composition. Solid lines are per eq 2 in the stick limit for each chromophore, and dashed lines denote eq 2-calculated slip-limit values for each fluorophore. Data points are the average of six measurements for each sample and the uncertainties are ±1σ.

For the ChCl:EG DESs investigated here, the bulk viscosities of these mixtures are known to depend on their composition.^22^ The experimental data for ChCl-EG DESs in the range of 1:2 through 1:6 are reported (Supporting Information of ref (21)), and those data can be fitted empirically to the function (Figure 2):

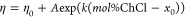
3where *η*_*0*_ = 13.31 cP, *A* = 4.426 cP, *x*_*0*_ = 0.7725, and *k* = 7.28 × 10^–2^. We emphasize that this is an empirical function and is not intended to represent a model based on physical reality. This function is used solely to extrapolate the viscosities of the ChCl:EG DES system to additional compositions with the recognition that the viscosity of any system is expected to be a continuous function of composition. With this caveat in mind, we can calculate the expected DSE model values for *τ*_*OR*_ for each chromophore in the stick and slip limits, as shown in Figure 3.

The data in Figure 3 reveal several interesting features. The first and perhaps most obvious feature is the substantial viscosity independence of the reorientation data for the chromophores perylene and Ox725. The viscosity over the DES composition range accessed varies by a factor of 3, and the viscosity near-independence for Ox725 and perylene indicates that the environment(s) sensed by the cationic (Ox725) and neutral, nonpolar (perylene) chromophores is not well-described by the bulk viscosity of the DES. The use of solvent bulk viscosity in eq 2 is an approximation because the interactions between the chromophore and its local environment are not the same chemically as the interactions between the solvent constituents. That said, there is a wealth of literature that shows experimental reorientation time constants to be proportional to solvent bulk viscosity.^43−45^ The fact that two of the chromophores we have examined exhibit substantially viscosity-independent behavior suggests that they are confined within an environment that is decoupled from that which determines the bulk behavior of the ChCl:EG DES system. It is also useful to note that the reorientation data for Ox725 and perylene exhibit time constants that are intermediate between the stick and slip limits, suggesting modest interactions, in energetic terms, between the chromophores and their local environments. These findings need to be viewed, however, in the context of systems that are not well-described by the modified DSE model, so there is limited information available from such comparisons for these two chromophores. In contrast, the data for DSF appear to be described reasonably well by the DSE stick-limit model. While the agreement does not follow the DSE model exactly, there is clearly a stronger viscosity dependence to these data. DSF is a dianion and, as such, is expected to interact differently with the ChCl:EG system than either a cationic (Ox725) or neutral (perylene) chromophore. These findings are consistent with what is known about the relative energetics of H-bonding in DESs (*vide infra*).

While the composition dependence of the DES system under consideration here has not been examined using rotational diffusion dynamics to this point, there have been reports on the reorientation dynamics of neutral and charged chromophores in some related DES systems.^23,46^ Hossain and Samanta reported on ethaline (1 ChCl:2 EG) using a neutral probe (coumarin 153), a cationic probe (rhodamine 123), and an anionic probe (fluorescein) and focusing on the temperature dependence of chromophore rotational diffusion. They found that, for this specific DES, coumarin 153 exhibited behavior that was intermediate between stick and slip limits, while the charged chromophores both showed stick-limit behavior. These data are not inconsistent with our findings and do indeed point to the importance of chromophore charge in interactions with their surroundings. The difference between the data reported by Hossain and Samanta^23^ and what we report here is that we consider changes in the DES system composition at fixed temperature, whereas they consider temperature-dependent changes in a specific DES composition. The viscosity dependence examined in their work rests on changes in the thermal energy within the system, while the viscosity dependencies we report here are a consequence of changes in the molecular composition and organization of the chromophore local environments independent of thermal changes.

Work by Turner and Kim focused on the spectral relaxation dynamics of coumarin 153 in selected DESs, providing insight into how the identity of the H-bond-donating constituent in the DES influences the local environment.^46^ Rotational diffusion measurements pointed to viscosity-dependent behavior intermediate between stick- and slip-limit dynamics and the so-called solvation times reported also scaled with viscosity to a fractional power. These authors reported different behavior for excitation of the chromophore on the red edge of the absorption band, suggesting long-lived, low-energy local environments, consistent with the established heterogeneous nature of DESs.

There is a significant body of literature that covers the organization and dynamics of DESs and, in particular, the ChCl:EG system.^21,47−50^ In such studies, the combination of scattering (neutron and X-ray) data and computational (MD) methods has provided insight into the organization of the ChCl:EG system; as such, it is tempting to invoke “structures” containing the hydrogen bond donor and acceptor constituents. We believe that the presentation of such “structures” may be somewhat misleading because DESs are liquid phase systems and fluid dynamics must therefore contribute significantly to the observed bulk properties of these systems. That said, it is important to note that, for ethaline, Zhang et al. provide information on the lifetimes of different hydrogen bonds in the ChCl-EG systems studied based on classical molecular dynamics (CMD) simulations.^21^ These lifetimes are important pieces of information because they are related to the average energy of each type of interaction. In that work, they report a time constant τ for the choline-Cl^–^ hydrogen bond of *ca*. 1460 ps and for the EG-Cl^–^ hydrogen bond of *ca*. 970 ps.^21^ If we take the strength of the hydrogen bond in EG (EG-EG; time constant, 32 ps) to be 5.5 kcal/mol^51^ and use that value to determine the effective Arrhenius prefactor for the ChCl:EG system,
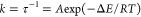
4

*A* = 3.37 × 10^14^, and the time constants for the long-lived interactions between choline and Cl^–^ and between EG and Cl^–^ yield H-bond energies of *ca*. 7.5 to 7.8 kcal/mol. This difference of *ca*. 2 kcal/mol for H-bonding interactions with the Cl^–^ anion suggests that such H-bonding interactions with anions dominate the structures relevant to determining DES behavior. The fact that we measure stronger interactions between DSF and the ChCl:EG system than we see for the neutral and cationic chromophores is consistent with the CMD results.^21^ The basis for this statement is that DSF, being negatively charged, and doubly so in fact, may be able to participate in the formation of DES organization more directly than the cationic or neutral chromophores, and because of such participation, DSF would sense the local environment on a length scale more commensurate with the entities that are responsible for the bulk viscosity of the DES. In this interpretation, the DSF dianion exists in an environment in which it is an active participant, a departure from that for the neutral and cationic chromophores. Of relevance, we note that strong interactions between the fluorescein chromophore and polyols have been reported previously.^52^

As noted above, the three chromophores exhibit rotational diffusion behavior that depends on the charge of the chromophore, and we understand this in the context of interactions between the chromophores and the inherently heterogeneous local organization known to exist in DESs. While the viscosity dependencies of the τ_OR_ data are as indicated above, a closer examination of the rotational diffusion data reveals, however, a subtle similarity between the three chromophores. That is, for all three chromophores, there exists a nonmonotonic trend in the composition-dependent reorientation time constants, with the change in behavior occurring between 10 and 15 mol % ChCl, as shown in Figure 3. This finding invites comparison to recent results from the Sangoro group pointing to composition-dependent dynamics in ChCl:EG DESs.^28^ In that work, the authors found that ChCl:EG DESs in the range of 15–20 mol % ChCl exhibited local minima or maxima in a variety of physical properties (e.g., dc ionic conductivity), evidencing enhanced rotational dynamics and charge transport that the authors use as evidence to suggest this to be the true eutectic composition. We believe that the differences sensed by the anion appear to be more pronounced than for the neutral or cation fluorophores for the same basic reason that the anion exhibits a more prominent viscosity dependence. That is, the anion is a better sensor of the composition-dependent changes in the DES system on the molecular scale, and the cation and anion chromophores sense region(s) that experience less microviscosity change with composition. All of this points to the heterogeneous nature of this DES system.

**Figure 4:**
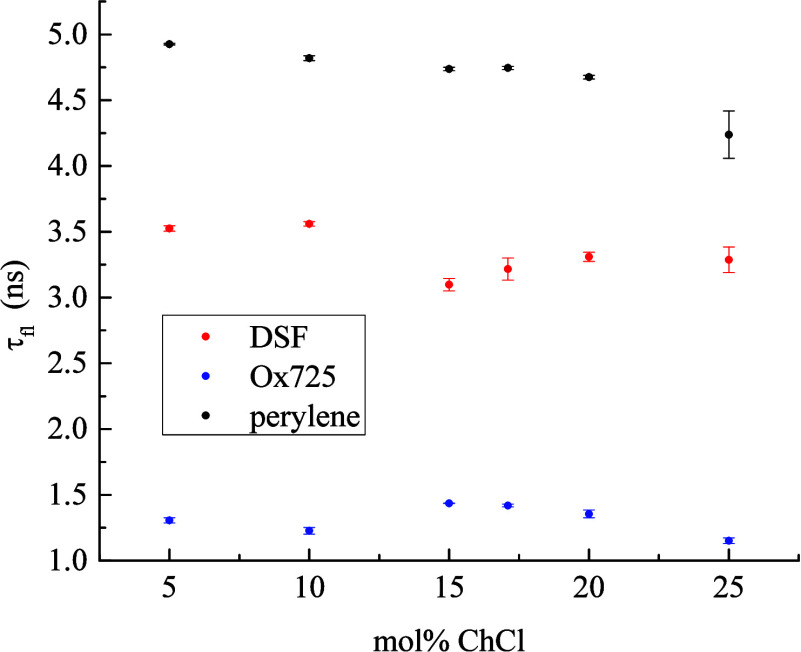
τ_fl_ as a function of DES composition. While the dependence of fluorescence lifetime on DEC composition is modest, there is a subtle variation for all three chromophores in the compositional region between 10 and 15 mol % ChCl. Data points are the average of six measurements for each sample and the uncertainties are ±1σ.

It is also of interest to examine the local environment of each chromophore through the fluorescence lifetime (τ_fl_). While there is not a well-established framework for the interpretation of τ_fl_ data in the same sense that there is for τ_OR_ data, it is important to determine whether any specific intermolecular interactions have a measurable effect on nonradiative decay channels, and it is known that the fluorescence lifetime of a chromophore depends on the dielectric response of its immediate environment.^53−57^ We show the data for τ_fl_ as a function of ChCl:EG DES composition in Figure 4. These data reveal no major changes in the fluorescence lifetime, but for each chromophore, there is a subtle change in the τ_fl_ composition dependence in the same composition range as for the τ_OR_ data (Figure 3). This finding is a further indication that the local environment of each chromophore varies with composition. Not surprisingly, the dependence is least for the nonpolar chromophore perylene and is more pronounced for the charged chromophores. It is also interesting to note that the composition dependence of τ_fl_ for DSF changes in a manner that is different than for Ox725, underscoring the difference in the local environments of the charged chromophores, although it is not possible to extract more detailed structural information from these data.

Although there are several different physical phenomena that are shown to exhibit such behavior, there is little in the way of detailed chemical insight available from these data. In many respects, this work raises more questions than it resolves. However, given the limited understanding of dynamics within DESs, we consider this a significant step forward. For example, these data do suggest that there is a stoichiometric relationship between ChCl and EG that gives rise to optimal interactions, at least from a dynamics point of view. Unfortunately, the reorientation data for the chromophores we report on here do not provide detailed insight into what the optimal organization might be. The next logical step in these studies is to investigate the specific local organization of the probe environments, as well as to determine the degree of heterogeneity in microscopic environments. Previous studies by the Meuwly group have successfully investigated these phenomena using 2D-IR and THz methods coupled to MD simulations on a KSCN/acetamide DES system.^58^ This work has provided substantial insight into heterogeneity using the KSCN probe, and it is expected that the use of organic fluorophores as probes may provide complementary but not identical results, owing to the likely active role that some probes may play in mediating DES local organization (*vide infra*). It also remains to be seen whether these observations are universal for similar canonical binary DES systems (e.g., ChCl:glycerol). Additionally, our results suggest that higher-valency probes, such as the trianionic 8-hydroxypyrene-1,3,6-trisulfonate (pyranine) species, might be particularly useful for tracking longer-scale DES dynamics or organization.^59^

## Conclusions

We have examined systematically the rotational diffusion dynamics of three chromophores of varying charge and polarity in a series of ChCl:EG DES compositions. The neutral and cationic chromophores exhibit behavior that is largely independent of DES bulk viscosity, while the anionic chromophore exhibits behavior that is described qualitatively in the context of the stick-limit DSE model. The chromophore charge-dependent dynamics is consistent with X-ray and neutron scattering studies and molecular dynamics calculations that point to the higher interaction energies associated with both the hydrogen bond donor (EG) and hydrogen bond acceptor (Ch^+^) interactions with chloride in the DES. These data point to the central role that the anionic species play in mediating DES behavior, and these findings may be consistent with the observed DES composition-dependent dynamics. Our reorientation data also appear to point to a region around 15 mol % ChCl where the stoichiometric relationship between the species is optimal. Further experimentation will be required to address the details of the molecular-scale organization in this composition range.

## Supporting Information Available

Tables of DES system compositions and composition-dependent fluorescence lifetime and reorientation time (PDF)

## References

[ref1] Ma Y.; Fu S.; Gao S.; Zhang S.; Che X.; Wang Q.; Jiao Z. Update on volatile organic compound (VOC) source profiles and ozone formation potential in synthetic resins industry in China. Environ. Pollut. 2021, 291, 118253 10.1016/j.envpol.2021.118253. 34597734

[ref2] Zhu B.; Zhong X.; Cai W.; Shi C.; Shao X.; Chen Z.; Yang J.; Chen Y.; Ni E.; Guo S.; Man H.; et al. Characterization of VOC source profiles, chemical reactivity, and cancer risk associated with petrochemical industry processes in Southeast China. Atmos. Environ: X 2024, 21, 100236 10.1016/j.aeaoa.2024.100236.

[ref3] Wang H.; Nie L.; Li J.; Wang Y.; Wang G.; Wang J.; Hao Z. Characterization and assessment of volatile organic compounds (VOCs) emissions from typical industries. Chin. Sci. Bull. 2013, 58 (7), 724–730. 10.1007/s11434-012-5345-2.

[ref4] Smith E. L.; Abbott A. P.; Ryder K. S. Deep Eutectic Solvents (DESs) and Their Applications. Chem. Rev. 2014, 114, 11060–11082. 10.1021/cr300162p. 25300631

[ref5] Zhang Q.; De Oliveira Vigier K.; Royer S.; Jérôme F. Deep eutectic solvents: syntheses, properties and applications. Chem. Soc. Rev. 2012, 41, 7108–7146. 10.1039/c2cs35178a. 22806597

[ref6] Zhao D.; Liao Y.; Zhang Z. Toxicity of Ionic Liquids. CLEAN – Soil, Air, Water 2007, 35, 42–48. 10.1002/clen.200600015.

[ref7] Docherty K. M.; Kulpa C. F. Jr. Toxicity and antimicrobial activity of imidazolium and pyridinium ionic liquids. Green Chem. 2005, 7, 185–189. 10.1039/b419172b.

[ref8] Kalhor P.; Ghandi K. Deep Eutectic Solvents as Catalysts for Upgrading Biomass. Catalysts 2021, 11, 178–210. 10.3390/catal11020178.

[ref9] Palmelund H.; Andersson M. P.; Asgreen C. J.; Boyd B. J.; Rantanen J.; Löbmann K. Tailor-made solvents for pharmaceutical use? Experimental and computational approach for determining solubility in deep eutectic solvents (DES). Int. J. Pharm: X 2019, 1, 100034 10.1016/j.ijpx.2019.100034. 31993583 PMC6977171

[ref10] Liao H.-G.; Jiang Y.-X.; Zhou Z.-Y.; Chen S.-P.; Sun S.-G. Shape-Controlled Synthesis of Gold Nanoparticles in Deep Eutectic Solvents for Studies of Structure–Functionality Relationships in Electrocatalysis. Angew. Chem., Int. Ed. Engl. 2008, 47, 9100–9103. 10.1002/anie.200803202. 18925592

[ref11] Wagle D. V.; Zhao H.; Baker G. A. Deep Eutectic Solvents: Sustainable Media for Nanoscale and Functional Materials. Acc. Chem. Res. 2014, 47, 2299–2308. 10.1021/ar5000488. 24892971

[ref12] Alonso D. A.; Baeza A.; Chinchilla R.; Guillena G.; Pastor I. M.; Ramón D. J. Deep Eutectic Solvents: The Organic Reaction Medium of the Century. Eur. J. Org. Chem. 2016, 2016, 612–632. 10.1002/ejoc.201501197.

[ref13] Ghosh S. K.; Nagarajan R. Deep eutectic solvent mediated synthesis of quinazolinones and dihydroquinazolinones: synthesis of natural products and drugs. RSC Adv. 2016, 6 (33), 27378–27387. 10.1039/C6RA00855K.

[ref14] Ruß C.; König B. Low melting mixtures in organic synthesis – an alternative to ionic liquids?. Green Chem. 2012, 14, 2969–2982. 10.1039/c2gc36005e.

[ref15] Ishtaweera P.; Ray C. L.; Filley W.; Cobb G.; Baker G. A. Nanoplastics Extraction from Water by Hydrophobic Deep Eutectic Solvents. ACS Appl. Eng. Matls. 2024, 2, 1460–1466. 10.1021/acsaenm.4c00159.

[ref16] Imperato G.; Eibler E.; Niedermaier J.; König B. Low-melting sugar–urea–salt mixtures as solvents for Diels–Alder reactions. Chem. Commun. 2005, 1170–1172. 10.1039/B414515A. 15726181

[ref17] Dai Y.; van Spronsen J.; Witkamp G.-J.; Verpoorte R.; Choi Y. H. Natural deep eutectic solvents as new potential media for green technology. Ana. Chim. Acta 2013, 766, 61–68. 10.1016/j.aca.2012.12.019. 23427801

[ref18] Gore S.; Baskaran S.; Koenig B. Efficient synthesis of 3,4-dihydropyrimidin-2-ones in low melting tartaric acid–urea mixtures. Green Chem. 2011, 13, 1009–1013. 10.1039/c1gc00009h.

[ref19] Abbott A. P.; Capper G.; Davies D. L.; Rasheed R. K.; Tambyrajah V. Novel solvent properties of choline chloride/urea mixtures. Chem. Commun. 2003, 1, 70–71. 10.1039/b210714g. 12610970

[ref20] Francisco M.; van den Bruinhorst A.; Kroon M. C. Low-Transition-Temperature Mixtures (LTTMs): A New Generation of Designer Solvents. Angew. Chem., Int. Ed. Engl. 2013, 52, 3074–3085. 10.1002/anie.201207548. 23401138

[ref21] Zhang Y.; Poe D.; Heroux L.; Squire H.; Doherty B. W.; Long Z.; Dadmun M.; Gurkan B.; Tuckerman M. E.; Maginn E. J. Liquid Structure and Transport Properties of the Deep Eutectic Solvent Ethaline. J. Phys. Chem. B 2020, 124, 5251–5264. 10.1021/acs.jpcb.0c04058. 32464060

[ref22] Gajardo-Parra N. F.; Cotroneo-Figueroa V. P.; Aravena P.; Vesovic V.; Canales R. I. Viscosity of Choline Chloride-Based Deep Eutectic Solvents: Experiments and Modeling. J. Chem. Eng. Data 2020, 65, 5581–5592. 10.1021/acs.jced.0c00715.

[ref23] Hossain S. S.; Samanta A. Solute Rotation and Translation Dynamics in an Ionic Deep Eutectic Solvent Based on Choline Chloride. J. Phys. Chem. B 2017, 121, 10556–10565. 10.1021/acs.jpcb.7b08472. 29087713

[ref24] Boogaart D. J.; Essner J. B.; Baker G. A. Evaluation of canonical choline chloride based deep eutectic solvents as dye-sensitized solar cell electrolytes. J. Chem. Phys. 2021, 155, 061102 10.1063/5.0055644. 34391350

[ref25] Hansen B. B.; Spittle S.; Chen B.; Poe D.; Zhang Y.; Klein J. M.; Horton A.; Adhikari L.; Zelovich T.; Doherty B. W.; et al. Deep Eutectic Solvents: A Review of Fundamentals and Applications. Chem. Rev. 2021, 121, 1232–1285. 10.1021/acs.chemrev.0c00385. 33315380

[ref26] Agieienko V.; Buchner R. Is ethaline a deep eutectic solvent?. Phys. Chem. Chem. Phys. 2022, 24, 5265–5268. 10.1039/D2CP00104G. 35171191

[ref27] Agieienko V.; Neklyudov V.; Buchner R. Why Does Ethaline Apparently Behave as an Ideal Binary Mixture?. J. Phys. Chem. Lett. 2022, 13, 10805–10809. 10.1021/acs.jpclett.2c02901. 36375079

[ref28] Spittle S.; Alfurayj I.; Hansen B. B.; Glynn K.; Brackett W.; Pandian R.; Burda C.; Sangoro J. Enhanced Dynamics and Charge Transport at the Eutectic Point: A New Paradigm for the Use of Deep Eutectic Solvent Systems. JACS Au 2023, 3, 3024–3030. 10.1021/jacsau.3c00420. 38034979 PMC10685424

[ref29] Hossain S. S.; Samanta A. Solute rotation and solvation dynamics in deep eutectic solvents. Chem. Phys. Impact 2021, 3, 100043 10.1016/j.chphi.2021.100043.

[ref30] Pal T.; Biswas R. Heterogeneity and viscosity decoupling in (acetamide+electrolyte) molten mixtures: A model simulation study. Chem. Phys. Lett. 2011, 517, 180–185. 10.1016/j.cplett.2011.11.002.

[ref31] Das A.; Biswas R. Dynamic Solvent Control of a Reaction in Ionic Deep Eutectic Solvents: Time-Resolved Fluorescence Measurements of Reactive and Nonreactive Dynamics in (Choline Chloride + Urea) Melts. J. Phys. Chem. B 2015, 119, 10102–10113. 10.1021/acs.jpcb.5b04936. 26159658

[ref32] Das S.; Biswas R.; Mukherjee B. Orientational Jumps in (Acetamide + Electrolyte) Deep Eutectics: Anion Dependence. J. Phys. Chem. B 2015, 119, 11157–11168. 10.1021/acs.jpcb.5b03022. 26131593

[ref33] Mukherjee K.; Das A.; Choudhury S.; Barman A.; Biswas R. Dielectric Relaxations of (Acetamide + Electrolyte) Deep Eutectic Solvents in the Frequency Window, 0.2 ≤ ν/GHz ≤ 50: Anion and Cation Dependence. J. Phys. Chem. B 2015, 119, 8063–8071. 10.1021/acs.jpcb.5b01502. 26012789

[ref34] Guchhait B.; Al Rasid Gazi H.; Kashyap H. K.; Biswas R. Fluorescence Spectroscopic Studies of (Acetamide + Sodium/Potassium Thiocyanates) Molten Mixtures: Composition and Temperature Dependence. J. Phys. Chem. B 2010, 114, 5066–5081. 10.1021/jp1001176. 20345185

[ref35] Smith C. J. II; Wagle D. V.; Bhawawet N.; Gehrke S.; Hollóczki O.; Pingali S. V.; O’Neill H.; Baker G. A. Combined Small-Angle Neutron Scattering, Diffusion NMR, and Molecular Dynamics Study of a Eutectogel: Illuminating the Dynamical Behavior of Glyceline Confined in Bacterial Cellulose Gels. J. Phys. Chem. B 2020, 124, 7647–7658. 10.1021/acs.jpcb.0c04916. 32790399

[ref36] Boogaart D. J.; Baker G. A. Moving Beyond Choline: Protic Choline Iodide Analogues toward Cosolvent-Free, Self-Contained Deep Eutectic Electrolytes for Dye-Sensitized Solar Cells. ACS Appl. Eng. Matls. 2024, 2, 360–367. 10.1021/acsaenm.3c00673.

[ref37] Pillman H. A.; Blanchard G. J. Effects of Energy Dissipation on Motional Dynamics in Unilamellar Vesicles. J. Phys. Chem. B 2010, 114, 13703–13709. 10.1021/jp1045723. 20942437

[ref38] Wagle D. V.; Deakyne C. A.; Baker G. A. Quantum Chemical Insight into the Interactions and Thermodynamics Present in Choline Chloride Based Deep Eutectic Solvents. J. Phys. Chem. B 2016, 120, 6739–6746. 10.1021/acs.jpcb.6b04750. 27268431

[ref39] Debye P. Polar Molecules; Chemical Catalog Company, Inc., 1929.

[ref40] Edward J. T. Molecular volumes and the Stokes-Einstein equation. J. Chem. Educ. 1970, 47, 261–270. 10.1021/ed047p261.

[ref41] Perrin F. Mouvement brownien d’un ellipsoide - I. Dispersion diélectrique pour des molécules ellipsoidales. J. Phys. Radium 1934, 5, 497–511. 10.1051/jphysrad:01934005010049700.

[ref42] Hu C.-M.; Zwanzig R. Rotational friction coefficients for spheroids with the slipping boundary condition. J. Chem. Phys. 1974, 60, 4354–4357. 10.1063/1.1680910.

[ref43] Steele W. A. Molecular Reorientation in Liquids. I. Distribution Functions and Friction Constants. J. Chem. Phys. 1963, 38, 2404–2410. 10.1063/1.1733516.

[ref44] Williams A. M.; Jiang Y.; Ben-Amotz D. Molecular reorientation dynamics and microscopic friction in liquids. Chem. Phys. 1994, 180, 119–129. 10.1016/0301-0104(93)E0421-Q.

[ref45] Zhang Y.; Venable R. M.; Pastor R. W. Molecular Dynamics Simulations of Neat Alkanes: The Viscosity Dependence of Rotational Relaxation. J. Phys. Chem. 1996, 100, 2652–2660. 10.1021/jp952745a.

[ref46] Turner A. H.; Kim D. Rotation and translation dynamics of coumarin 153 in choline chloride-based deep eutectic solvents. J. Chem. Phys. 2018, 149, 174503 10.1063/1.5038067. 30409002

[ref47] Bryant S. J.; Christofferson A. J.; Greaves T. L.; McConville C. F.; Bryant G.; Elbourne A. Bulk and interfacial nanostructure and properties in deep eutectic solvents: Current perspectives and future directions. J. Colloid Interface Sci. 2022, 608, 2430–2454. 10.1016/j.jcis.2021.10.163. 34785053

[ref48] Kaur S.; Kumari M.; Kashyap H. K. Microstructure of Deep Eutectic Solvents: Current Understanding and Challenges. J. Phys. Chem. B 2020, 124, 10601–10616. 10.1021/acs.jpcb.0c07934. 33151072

[ref49] LaRocca M. M.; Baker G. A.; Heitz M. P. Assessing rotation and solvation dynamics in ethaline deep eutectic solvent and its solutions with methanol. J. Chem. Phys. 2021, 155, 034505 10.1063/5.0056653. 34293899

[ref50] Wagle D. V.; Zhao H.; Deakyne C. A.; Baker G. A. Quantum Chemical Evaluation of Deep Eutectic Solvents for the Extractive Desulfurization of Fuel. ACS Sus. Chem. Eng. 2018, 6, 7525–7531. 10.1021/acssuschemeng.8b00224.

[ref51] Olsen R.; Kvamme B.; Kuznetsova T. Hydrogen bond lifetimes and statistics of aqueous mono-, di- and tri-ethylene glycol. AIChE J. 2017, 63, 1674–1689. 10.1002/aic.15539.

[ref52] Feldman H.; Iron M. A.; Fixler D.; Moshkov S.; Zurgil N.; Afrimzon E.; Deutsch M. Fluorophore spectroscopy in aqueous glycerol solution: the interactions of glycerol with the fluorophore. Photochem. Photobio. Sci. 2021, 20, 1397–1418. 10.1007/s43630-021-00096-w. 34609728

[ref53] Drexhage K. H. Influence of a dielectric interface on fluorescence decay time. J. Lumin. 1970, 1 (2), 693–701. 10.1016/0022-2313(70)90082-7.

[ref54] Girard C.; Martin O. J. F.; Dereux A. Molecular lifetime changes induced by nanometer scale optical fields. Phys. Rev. Lett. 1995, 75, 3098–3101. 10.1103/PhysRevLett.75.3098. 10059494

[ref55] Lukosz W.; Kunz R. E. Fluorescence lifetime of magnetic and electric dipoles near a dielectric interface. Opt. Commun. 1977, 20, 195–199. 10.1016/0030-4018(77)90331-5.

[ref56] Lukosz W.; Kunz R. E. Light emission by magnetic and electric dipoles close to a plane interface. I. Total radiated power. J. Opt. Soc. Am. 1977, 67, 1607–1619. 10.1364/JOSA.67.001607.

[ref57] Cucchi M.; Matulaitis T.; Kukhta N. A.; Grazulevicius J. V.; Reineke S.; Scholz R. Influence of the Dielectric Constant around an Emitter on Its Delayed Fluorescence. Phys. Rev. Appl. 2019, 12, 044021 10.1103/PhysRevApplied.12.044021.

[ref58] Töpfer K.; Pasti A.; Das A.; Salehi S. M.; Vazquez-Salazar L. I.; Rohrbach D.; Feurer T.; Hamm P.; Meuwly M. Structure, Organization, and Heterogeneity of Water-Containing Deep Eutectic Solvents. J. Am. Chem. Soc. 2022, 144, 14170–14180. 10.1021/jacs.2c04169. 35895323

[ref59] Yung K. Y.; Schadock-Hewitt A. J.; Hunter N. P.; Bright F. V.; Baker G. A. ‘Liquid litmus’: chemosensory pH-responsive photonic ionic liquids. Chem. Commun. 2011, 47, 4775–4777. 10.1039/c1cc00065a. 21399813

