# Supplementary material for: Molecular-Scale Interactions in the Choline Chloride–Ethylene Glycol Deep Eutectic Solvent System: The Importance of Chromophore Charge in Mediating Rotational Dynamics
Source: J Phys Chem B. 2024 Sep 24;128(39):9536–43. doi: 10.1021/acs.jpcb.4c04118 (PMC11457140; doi:10.1021/acs.jpcb.4c04118)
Supplement: Supplementary file 1 — jp4c04118_si_001.pdf [file jp4c04118_si_001.pdf]

Supporting Information

Molecular-Scale Interactions in the Choline Chloride-Ethylene Glycol Deep Eutectic Solvent System. The Importance of Chromophore Charge in Mediating Rotational Dynamics

Allison Stettler[1], Piyuni Ishtaweera[2], Gary A. Baker[2] and G. J. Blanchard[1,*]

[1] Michigan State University, Department of Chemistry, East Lansing, MI USA 48824-1322

[2] University of Missouri-Columbia, Department of Chemistry, Columbia, MO USA 65211

Table S1. ChCl:EG Samples Spanning the 5–33 mol% ChCl Range.

mol% ChCl	mol% EG	ChCl:EG ratio	ChCl (g)	EG (g)
5	95	1:19	10	88
10	90	1:9	21	84
15	85	1:5.67	31	79
17.1	82.9	1:4.85	36	77
20	80	1:4	42	74
25	75	1:3	52	70
33 ⅓	66 ⅔	1:2	70	62

* Author to whom correspondence should be addressed. email blanchard@chemistry.msu.edu, Tel: +1 517 353 1105.

Table S2. Fluorescence lifetimes, zero-time anisotropy and reorientation times for perylene, oxazine 725 and disodium fluorescein as a function of DES composition. Data values are the average of six measurements for each sample and the uncertainties are ± 1σ.

	Perylene		Oxazine 725		Disodium fluorescein	
**ChCl (mol%)**	**τ_{fl} (ps)**	**τ_{OR} (ps)**	**τ_{fl} (ps)**	**τ_{OR} (ps)**	**τ_{fl} (ps)**	**τ_{OR} (ps)**
5	4926±6	484±58	1306±19	891±43	3525±21	1362±43
10	4819±18	543±39	1227±25	939±25	3560±17	1242±82
15	4737±13	451±28	1436±2	1064±66	3098±47	1660±71
17.1	4746±12	482±43	1419±10	1036±79	3216±84	1791±67
20	4675±14	497±55	1355±30	1017±68	3310±35	1902±63
25	4283±180	542±74	1151±22	973±23	3287±97	2015±162
